# Quantum interference in heterogeneous superconducting-photonic circuits on a silicon chip

**DOI:** 10.1038/ncomms10352

**Published:** 2016-01-21

**Authors:** C. Schuck, X. Guo, L. Fan, X. Ma, M. Poot, H. X. Tang

**Affiliations:** 1 Department of Electrical Engineering, Yale University, New Haven, Connecticut 06511, USA; 2 Institute for Quantum Optics and Quantum Information, Austrian Academy of Science, A-1090 Vienna, Austria

## Abstract

Quantum information processing holds great promise for communicating and computing data efficiently. However, scaling current photonic implementation approaches to larger system size remains an outstanding challenge for realizing disruptive quantum technology. Two main ingredients of quantum information processors are quantum interference and single-photon detectors. Here we develop a hybrid superconducting-photonic circuit system to show how these elements can be combined in a scalable fashion on a silicon chip. We demonstrate the suitability of this approach for integrated quantum optics by interfering and detecting photon pairs directly on the chip with waveguide-coupled single-photon detectors. Using a directional coupler implemented with silicon nitride nanophotonic waveguides, we observe 97% interference visibility when measuring photon statistics with two monolithically integrated superconducting single-photon detectors. The photonic circuit and detector fabrication processes are compatible with standard semiconductor thin-film technology, making it possible to implement more complex and larger scale quantum photonic circuits on silicon chips.

## Abstract (web summary)

Scaling photonic quantum information processing approaches remains challenging for integrated quantum optics. Here, Schuck *et al.* develop a hybrid superconducting-photonic circuit system to show how quantum interference and single-photon detectors can be combined in a scalable fashion on a silicon chip.

## Graphical abstract

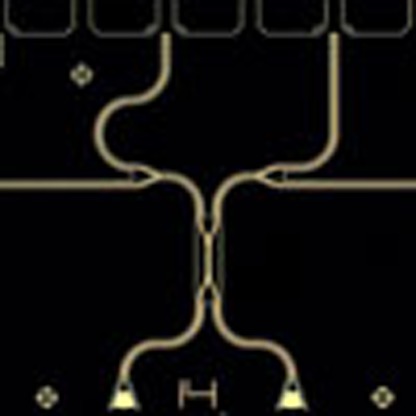

Proof-of-principle experiments have shown that quantum information processing has great potential for solving certain computational tasks, which are intractable with classical means^1^. Among the various approaches, integrated quantum photonics has emerged as a particularly interesting one for realizing optical quantum simulations^2^,^3^,^4^,^5^, quantum information processing^6^,^7^,^8^,^9^ and communication^10^,^11^. However, scaling current quantum technology to larger system sizes remains a significant challenge owing to the demanding requirements for high-fidelity signal processing at single-photon levels.

Advanced nanofabrication techniques have proven invaluable for ensuring scalability of electronic components used in classical information technology^12^. The corresponding complementary metal oxide semiconductor (CMOS) fabrication recipes have recently also been employed for realizing both nanophotonic waveguides^13^, as well as superconducting single-photon detectors (SSPD)^14^ on silicon chips. As most linear optics quantum logic schemes rely on non-classical interference and single-photon detection^15^,^16^ it is crucial to realize both of these ingredients on a common scalable platform. Here we demonstrate such a quantum information processing platform by combining SSPDs with integrated silicon nitride photonic circuits to measure high-visibility quantum interference directly on-chip.

Highly efficient single-photon detection has previously been achieved with fibre-coupled SSPDs^17^, which have found many exciting applications^18^,^19^,^20^. For integrated photonic technology, detection of photons inside a waveguide directly on-chip is required because it eliminates the chip-to-fibre interface, which is often a bottleneck in photonic device packaging. Optimal performance in this regard is achieved with nanowire SSPDs in travelling wave geometry^21^,^22^. This design is an excellent choice for integrated nanophotonic applications because large numbers of these compact detectors can be embedded directly in optical waveguide circuits^23^. On-chip detection efficiencies up to 90%^24^,^25^ (system detection efficiencies up to 10% (refs 26, 27)) at visible as well as telecom wavelengths have been demonstrated with such waveguide-coupled SSPDs. Furthermore, these detectors can operate at GHz rates, achieve <20 ps timing accuracy, sub-Hz dark count rate and extremely low-noise equivalent powers down to the 10^−20^ W Hz^−1/2^ level^24^,^28^.

Quantum interference can be observed when two indistinguishable photons impinge simultaneously on the inputs of a 50:50 beam splitter, that is, the probability of finding individual photons in separate output modes vanishes. This non-classical effect was first observed by Hong, Ou and Mandel (HOM)^29^ and is the consequence of destructive interference of the probability amplitudes corresponding to both photons being transmitted/reflected at the beam splitter^30^. A waveguide implementation of an optical beam splitter is an optimal choice for realizing spatial mode matching^31^,^32^,^33^,^34^, which is one of the limiting factors for achieving high-visibility quantum interference in free-space optics experiments.

The integration of photonic circuits and detectors on a silicon chip for demonstrating quantum interference has previously been attempted with surface plasmon polariton devices. Two-plasmon quantum interference with 93% visibility on a beam splitter has been demonstrated with off-chip detectors and for photons at visible wavelengths^35^, which are not compatible with existing optical communication networks. Notably, the integration of plasmonic directional couplers with superconducting detectors on the same chip proved challenging and reduced the interference contrast below the classical limit^36^.

Here we integrate low-noise niobium titanium nitride (NbTiN) nanowire SSPDs with dielectric silicon nitride (SiN) photonic circuits on a silicon chip. Using photons from spontaneous parametric down conversion (SPDC) we measure quantum interference with 97% visibility directly on-chip. Our circuit-detector approach is fully compatible with scalable, high-yield semiconductor microfabrication processes.

## Results

### Experimental set-up

**Figure 1:**
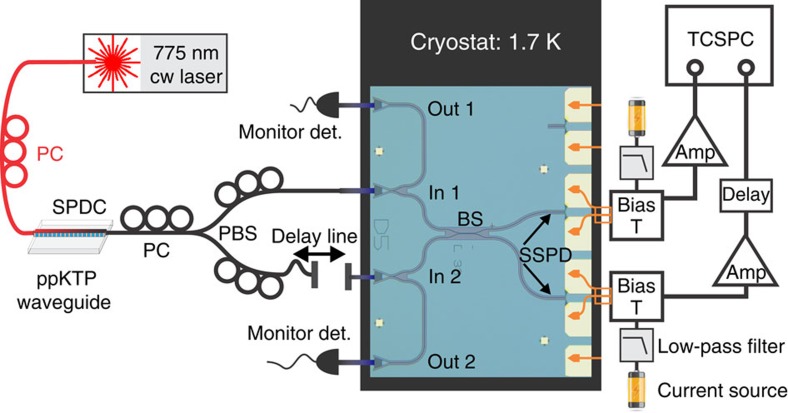
Schematic of the on-chip quantum interference measurement set-up. A 775-nm continuous wave (cw) diode laser is used as a pump for generating orthogonally polarized 1,550 nm photon pairs via type-II spontaneous parametric down conversion in a 10.5-mm-long fibre-coupled periodically poled KTP waveguide. Input polarizations to the ppKTP source and a polarizing fibre beam splitter (PBS) are adjusted with fibre polarization controllers (PC) for optimal SPDC efficiency and deterministic splitting of photon pairs into separate PBS-output modes, respectively. Temporal delay between photons in separate output modes is set with a fibre-free-space-fibre optical delay line. Light is then guided into a closed-cycle cryostat where it is coupled from an optical fibre array into on-chip SiN photonic circuits via optical grating couplers at In 1 and In 2 (optical micrograph). The alignment of the chip to the fibre array with low-temperature nanopositioners (attocube) is aided by monitoring the optical transmission at the auxiliary ports Out 1 and Out 2. Photons interfere at a 33-μm-long directional coupler (beam splitter, BS) with 400 nm gap between waveguides in the coupling region. SSPDs on top of the beam splitter's output waveguides are supplied with a 10–15 μA bias from a low-noise current source and read out via a rf-probe connected to a bias-T outside the cryostat. After signal amplification (PPL 5828 & RF-Bay LNA-4050), photon statistics are recorded with a time-correlated single-photon counting unit (PicoHarp 300).

The experimental set-up for measuring HOM interference with SSPDs directly on-chip is shown in Fig. 1. We produce energy–time correlated photon pairs via the process of type-II SPDC in a periodically poled potassium titanyl phosphate (ppKTP) crystal waveguide^37^ and couple them into nanophotonic waveguides on a silicon chip. A continuous wave 775 nm pump laser is coupled directly from an optical single-mode fibre into the ppKTP waveguide and the generated 1,550-nm photon pairs are collected into a single-mode fibre. In type-II SPDC the generated photons of one pair have orthogonal polarization. Hence we use a fibre polarization beam splitter (PBS) to deterministically separate photons of each pair. To do so, we optimize the efficiency of the source by using a 1,550 nm telecom laser, which we send via both inputs/outputs of the fibre PBS into the ppKTP waveguide, that is in reverse, and monitor the second harmonic generation (SHG) of 775 nm light. First we maximize the SHG power by optimizing the spatial alignment between the ppKTP crystal waveguide and the input/output optical fibres. Similarly we optimize the phase matching of the nonlinear process by adjusting the crystal temperature for pump light of a given wavelength and waveguide geometry. We then minimize the SHG power using only the polarization controller between the ppKTP crystal and the fibre PBS (Fig. 1), thus ensuring orthogonal polarizations (H/V) at the PBS. Switching back to the 775 nm pump laser we first adjust the polarization controllers between 775 nm laser and ppKTP waveguide to maximize the photon pair generation efficiency. Then we adjust the polarization controllers behind the fibre PBS such that we achieve optimal coupling to the transverse electric mode of the on-chip waveguides. We use a 1,064-nm long-pass and a 1,550-nm band-pass filter, which efficiently suppress 775 nm, pump light in combination with on-chip grating couplers, effectively acting as additional band-pass filters. We introduce an optical delay line in one of the output ports of the fibre PBS that allows us to scan the relative arrival time between two photons of a pair at the silicon chip.

**Figure 2:**
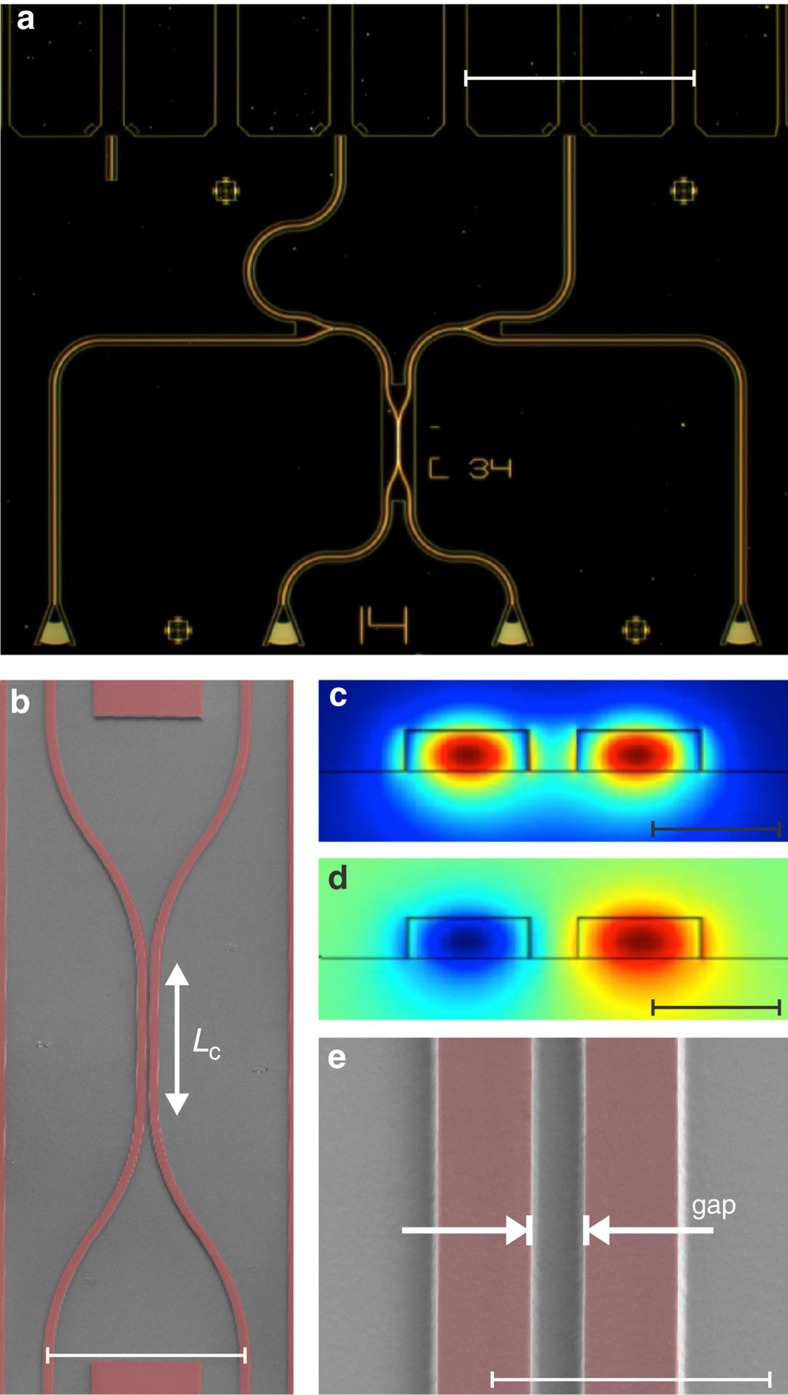
Directional coupler device design. (**a**) Dark field micrograph of a HOM device with two input grating couplers (bottom centre), two output grating couplers for device alignment and calibration (bottom left/right), the directional coupler (DC) and waveguide-coupled SSPDs, which are contacted via Au-electrode pads (top) (scale bar, 250 μm); (**b**) SEM image of a directional coupler of nominal length *L*_c_ made from 1-μm-wide SiN waveguides (scale bar, 25 μm); (**c**) finite element simulation of the symmetric transverse electric field mode in the coupling region (max./min.: 1.8 × 10^10^ Vm^−1^/0 Vm^−1^; scale bar, 1 μm); (**d**) anti-symmetric transverse electric mode (max./min.: ±1.8 × 10^10^ Vm^−1^; scale bar, 1 μm); (**e**) centre region of the directional coupler where evanescent coupling of the field modes occurs (scale bar, 3 μm).

The chip with ∼100 photonic integrated circuits and twice as many detectors (SSPDs) is mounted inside a closed-cycle cryostat, which provides continuous cooling to 1.7 K with <10 mK temperature variations^38^. We use a radiofrequency (rf) probe to make electrical contact to electrode pads on the chip, which connect to the SSPDs. The rf-probe lines are wired to a bias-T for supplying current from a low-noise source to the nanowires as well as reading out the voltage pulses upon photon detection by an SSPD^23^. Photons from the down conversion source are delivered to the on-chip photonic integrated circuits via an optical fibre array. Coupling loss from optical fibres to the on-chip waveguides is calibrated independently for each device input via monitor ports (Fig. 1) and is usually around 10 dB. The two device layouts shown in Figs 1 and 2a facilitate detector characterization, optical path-length measurements and fibre-to-device alignment. However, using 3 dB of the signal (idler) photons per input port for calibration purposes also leads to a quadratic decrease in the coincidence detection rates from correlated photon pairs. Future device designs could benefit from omitting these monitor ports. The chip is mounted on a stack of low-temperature compatible translation stages that allow us to position different devices under the fibre array and rf-probe-assembly for testing.

We amplify SSPD output signals before recording their arrival times with a time-correlated single-photon counting unit. In offline analysis we can thus identify detection events from correlated photon pairs by comparing arrival time lists for each of the two SSPDs^32^.

### Integrating photonic circuits and SSPDs

The device geometry for an on-chip HOM-interference measurement is shown in Fig. 2a. Fabrication starts from a commercial SiN on silicon dioxide (SiO_2_) on silicon (Si) wafer onto which we sputter a thin film of NbTiN. Subsequently we define electrode pads, nanowire detectors and waveguides in standard electron-beam lithography followed by lift off and dry-etching chemistry, respectively (see Methods section). We use optical grating couplers to transmit light from the optical fibre array into waveguides of 1 μm width, designed for transverse electric single-mode propagation on-chip^24^,^25^. Photons are then guided to a beam splitter and detected by NbTiN nanowire SSPDs at the beam splitter's output.

The beam splitter is implemented as a directional coupler where two input waveguides are brought into close proximity over a coupling length *L*_c_ (Fig. 2b). With the waveguides acting as polarizers and spatial mode filters, the photons are indistinguishable when they arrive at the directional coupler. Finite element simulations of the coupling region show evanescent coupling between transverse electric modes of 1-μm-wide SiN waveguides. The resulting symmetric (Fig. 2c) and anti-symmetric (Fig. 2d) hybrid modes have a refractive index difference of Δ*n* for a given gap. In simulations we find a coupling length of *L*_c_=*λ*/(4·Δ*n*)=28 μm or a 400-nm gap to realize 50:50 splitting between the 330 nm high waveguides (Fig. 2e). In calibration devices (Mach–Zehnder interferometers and beam splitters, see Supplementary Fig. 1 and Supplementary Note 1) we observe that a slightly larger coupler length of *L*_c_=33 μm is required to achieve a 50:50 splitting ratio, which accounts for waveguide asymmetry, width and gap-offsets, as well as additional coupling in the input and output region of the directional coupler, all of which are not taken into account in the finite element simulations. Owing to the high refractive index contrast of SiN on insulator (SiO_2_), the footprint of our devices is approximately two orders of magnitude smaller than glass-based waveguide implementations and could in principle be even more compact if a smaller gap is chosen (see Supplementary Note 1 and Supplementary Fig. 2).

**Figure 3:**
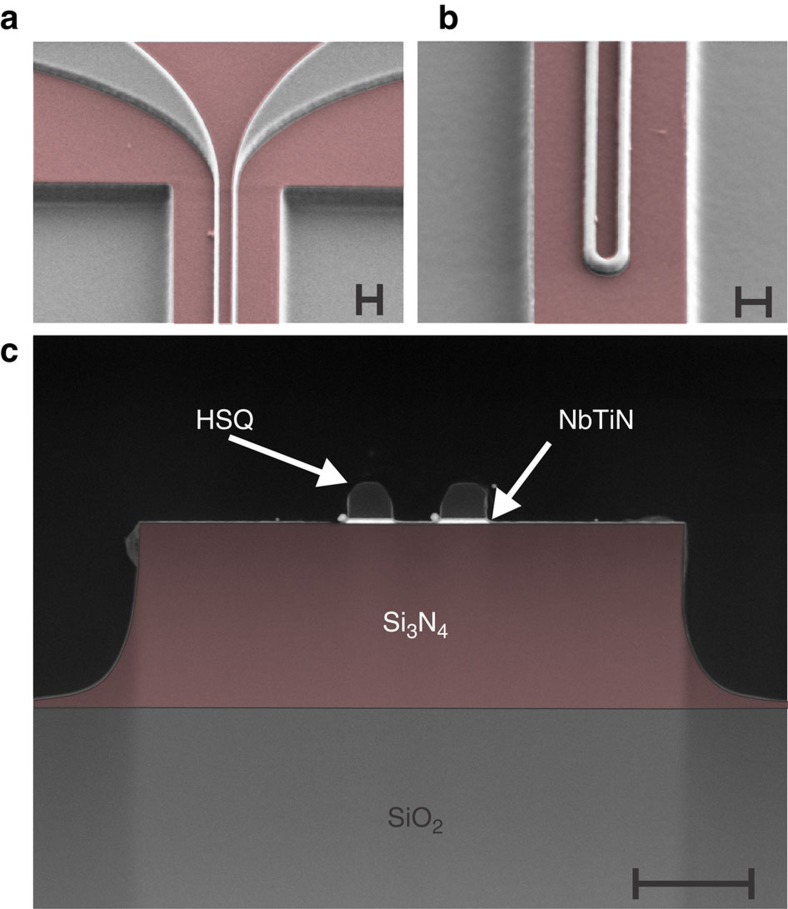
Waveguide-coupled SSPD. (**a**) Scanning electron micrograph of the leads connecting a 50-nm narrow nanowire SSPD to Au-contact pads (see Fig. 2a); (**b**) U-shaped part of a 40-μm-long nanowire SSPD for optimal bias current distribution in the bending region where photons are incident from the SiN-waveguide underneath; (**c**) transmission electron micrograph of the SSPD-waveguide cross-section. The 8-nm-thin NbTiN nanowire (here 80 nm wide) is covered with electron-beam lithography resist (hydrogen silsesquioxane, HSQ) and centred on top of a 330-nm-high SiN waveguide on a buried oxide layer. All scale bars, 200 nm.

SSPDs were fabricated from sputter deposited 8.2 nm thin NbTiN films (see Methods section). We pattern 50 nm wide, 40 μm long U-shaped nanowires, which are connected via wider leads (Fig. 3a) to the electrode pads (Fig. 2a). We ensure that the SSPDs are precisely centred on top of the waveguides for optimal performance by aligning the nanowires to the same marks, which are subsequently used for patterning the SiN layer. We confirm that layer alignment between different lithography steps is better than 50 nm in scanning and transmission electron microscopy, as shown in Fig. 3. A calibration of the detector performance yielded an on-chip detection efficiency of 11.5% and a dark count rate of 0.7 and 2.2 Hz for a typical pair of SSPDs, when biasing close to the critical current (including black body radiation and stray light). We observe that our fabrication process features high yield of functional devices (see Supplementary Note 2 and Supplementary Fig. 3).

### HOM-interference measurement

To measure quantum interference between photons produced in SPDC we first match the optical delays in the interferometer formed by the fibre PBS and the on-chip waveguide beam splitter (Fig. 1). We determine the zero-delay position using a 2.4-ps pulsed telecom wavelength laser (with corresponding 1.6 mm coherence length) and observing first-order interference between split pulses at one of the monitor ports with a fast O/E converter. Using this starting position we then send photon pairs from the SPDC source onto the chip and record detection events for both SSPDs at the outputs of the directional coupler.

**Figure 4:**
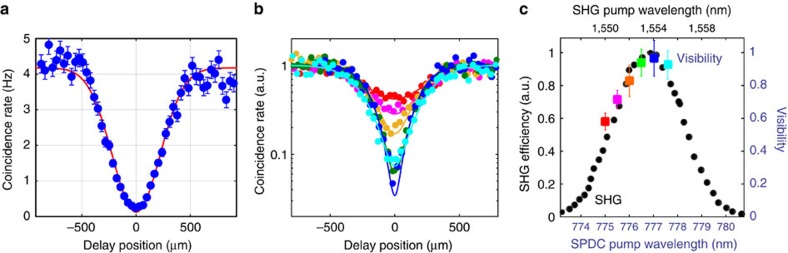
On-chip HOM interference. (**a**) Coincidence detection rate (raw) between SSPDs coupled to the directional coupler's output waveguides as a function of photon arrival time delay at a nominal pump power of 10.5 mW. Error bars show the s.d. of the statistical ensemble of coincidence events. As we scan the position of the optical delay line (Fig. 1) the coincidence rate drops from 4.2 Hz close to zero when photons created in SPDC arrive simultaneously at the on-chip directional coupler. From a Gaussian fit to the data we extract a two-photon interference visibility of *V=*0.97±0.05 and the coherence time of the SPDC photons as 1.7 ps; (**b**) measured coincidence rates as a function of relative delay between signal and idler photons for various SPDC pump laser wavelengths, *λ*_p_=775−777.6 nm. Error bars are similar to those shown in **a** but have been omitted for readability. From a fit to the data we find the visibilities *V*_red_(*λ*_p_=775 nm)=58±5%, *V*_magenta_(*λ*_p_=775.5 nm)=71±6%, *V*_orange_(*λ*_p_=776 nm)=83±9%, *V*_green_(*λ*_p_=776.5 nm)=94±8%, *V*_blue_(*λ*_p_=777.1 nm)=97±10%, *V*_cyan_(*λ*_p_=777.6 nm)=93±9%; (**c**) HOM-interference visibility as a function of SPDC pump wavelength as extracted from the fits in **b** where error bars denote 95% confidence bounds of the fit, and SHG efficiency as a function of pump wavelength at similar temperature of the ppKTP crystal.

For delay positions larger than the coherence length, *τ*_c_, of the down conversion photons we observe a coincidence rate of 4.2±0.1 Hz for 256 ps binning of the photon arrival time data using a nominal 775 nm pump power of 10.5 mW (before coupling to the ppKTP waveguide). As we scan the delay line around the zero-delay position we observe how the coincidence rate drops almost to zero from its initial value for unmatched arrival times at the beam splitter and then recovers to its initial value of ∼4.2 Hz away from the zero-delay position, as shown in Fig. 4a. This is the expected behaviour for HOM interference of temporally correlated indistinguishable photons.

We approximate the spectral bandwidth of the down conversion photons incident on the beam splitter by a Gaussian function and fit the raw coincidence rate data with the corresponding function^39^:


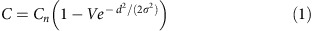


where *C*_*n*_ is the coincidence rate for photons with large arrival time delays 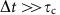, *V* is the HOM-interference visibility, *d* is the delay position and *σ* the standard deviation. From the fit shown in Fig. 4a we extract the visibility *V*=96.9±5.3% and a full width at half maximum of *w*=518±41 μm, corresponding to a coherence time of 1.7±0.1 ps from which we estimate a SPDC photon bandwidth of 2.1±0.2 nm (ref. 40).

Note that the HOM-interference visibility was extracted directly from the raw data shown in Fig. 4a. Hence it contains contributions from accidental coincidence detection events, where each SSPD registered a count within the user-specified coincidence time window, although no photon pair was detected. Such accidental coincidence events occur statistically as photons belonging to different pairs and/or detector dark counts, are registered within a time interval shorter than the coincidence time window. Here we choose a coincidence time window of 256 ps, which is significantly longer than the jitter of our SSPDs (∼50 ps (ref. 28)) but short enough to avoid a significant background of accidental coincidences: at <10 kHz counting rate per SSPD (depending on pump power) we estimate an accidental coincidence contribution of 0.02 Hz, which is contained in the rate shown in Fig. 4a. Our data clearly benefits from the low dark count rates of the two SSPDs used here (0.7 and 2.2 Hz, respectively), which only cause a negligible background contribution to the measured coincidence rate.

We repeat the two-photon interference measurement for a significantly lower pump power of 3.5 mW to avoid higher-order processes in the SPDC process but only observe a small improvement of HOM-interference visibility to 97.1% (see Supplementary Note 3 and Supplementary Fig. 4). We thus conclude that higher-order SPDC processes do not contribute appreciably to the observed coincidence rate at zero delay.

The fact that we observe a visibility slightly lower than 100% at *d*=0 is thus mainly due to detection events from independent pairs that were created within the coincidence detection window, a slight imbalance in the splitting ratio of our on-chip directional coupler and the statistical photon counting noise. We anticipate that fine tuning of our fabrication recipes will improve the performance of our photonic circuits and detectors to allow for even higher interference visibilities, which comply with fault-tolerant quantum operations^41^.

### Characterization of photon indistinguishability

To achieve optimal visibility in HOM interference it is necessary that signal and idler photons are indistinguishable at the beam splitter, not only in arrival time but also in all other degrees of freedom. In nanophotonic implementations, as the one presented here, the spatial overlap between the input modes of the directional coupler is guaranteed by the high lithographic control over the waveguide dimensions on-chip. For the waveguide cross-section chosen here (330 nm × 1 μm) only a single transverse electric mode is supported such that other polarization and spatial modes are efficiently suppressed. We thus investigate the remaining spectral distinguishability between the down conversion photons in dependence of SPDC pump detuning, which can be varied off-chip.

The frequency correlation between signal and idler output modes in SPDC is described by a joint spectral amplitude, which is given by the product of a pump spectral amplitude and a phase-matching function. The latter can be approximated by a Gaussian function with width *σ*_s_ around the degenerate (quasi) phase-matching frequency, *ω*_0_, where Δ**k**=0 with *ω*_s_=*ω*_i_. If the 775 nm pump frequency, *ω*_p_, is detuned from *ω*_0_, the signal and idler spectra around *ω*_s_ and *ω*_i_, respectively, will shift according to the phase-matching conditions imposed by energy and momentum conservation: *ω*_p_=*ω*_s_+*ω*_i_ and **Δk=k**_**p**_**−k**_**s**_**−k**_**i**_−2*π*/*Λ*, with poling period *Λ* and wave vectors 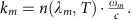 Here *T* is the SPDC source temperature and the refractive indices *n(λ*_*m*_,*T)* for pump, signal and idler photons, *m=*p,s,i, are given by the Sellmeier equations. A change in pump wavelength thus introduces spectral distinguishability between signal and idler photons, which reduces the visibility in a HOM-interference experiment.

We scan the delay between signal and idler photons around the zero-delay position for pump laser wavelengths of *λ*=775−777.6 nm and observe the variation of interference visibility with pump wavelength, shown in Fig. 4b. For better comparison all data is normalized to the coincidence rate at Δ*τ*→∞ as determined from the respective fit. At *λ*=775 nm pump wavelength the phase-matching conditions cause the signal and idler spectra to shift significantly such that the interference visibility drops to 58% (see red data in Fig. 4b). As the pump wavelength is increased to 777.1 nm the HOM-interference visibility gradually increases to 97% (see dark blue data in Fig. 4b) before it starts dropping again for *λ*>777.1 nm (see cyan data in Fig. 4b). The variation of interference visibility follows roughly a Gaussian distribution (Fig. 4c) as expected from shifting the approximately Gaussian signal and idler spectra with respect to each other. High visibility is achieved over a relatively broad spectral range (*V*>90% over Δ*λ*≈1.5 nm around *λ*=777.1 nm), which shows that signal and idler photon distinguishability is under accurate control in our experiment.

For comparison we show the SHG efficiency for pump wavelengths *λ*=1,546−1,562 nm of the ppKTP crystal at similar temperature in Fig. 4c. Both SHG efficiency and HOM-interference visibility show similar behaviour as a function of the respective pump laser wavelength. This relation between SHG and SPDC phase matching is expected in a crystal of given material properties, waveguide length and cross-section^42^.

## Discussion

Recent experiments in quantum optics^3^,^4^,^5^,^43^ manifest an ever more pressing need for a scalable solution to integrate photonic circuits and single-photon detectors. In particular, single-photon detection and high-visibility quantum interference have been identified as the two essential requirements for realizing scalable linear optic quantum computation^15^. Here we have shown how SSPDs embedded with nanophotonic circuits address these needs and achieve the requirements for scalable quantum technology on silicon chips. We observe high-visibility quantum interference of photons produced in SPDC with waveguide-coupled SSPDs and demonstrate that photon distinguishability is under accurate control in this architecture. All of the fabrication techniques used here can in principle be adapted to scalable technology developed for the CMOS industry, even at the front end of a CMOS line^44^. We anticipate that fine tuning of superconducting film and photonic circuit parameters and the implementation of photon-number resolving architectures^45^,^46^ will further increase the functionality of our integrated quantum photonic system.

Recent progress in realizing sources of non-classical light directly on silicon chips ideally complements the integration of single-photon detectors and photonic circuits described here. Such integrated quantum light sources were realized employing spontaneous four wave mixing^31^,^47^ and the excitation of waveguide-coupled quantum emitters^48^,^49^ but could also be realized via SPDC in III-nitride waveguides^50^,^51^. Combining nanophotonic sources, circuits and single-photon detectors on a silicon chip will allow for generating, processing and detecting quantum information all on one scalable platform.

## Methods

### Device fabrication

We deposit NbTiN on commercial 330 nm stoichiometric Si_3_N_4_ on 3 μm thermally grown SiO_2_ on Si wafers. The film thickness of the NbTiN layer is controlled via timed reactive ion sputtering from an NbTi alloy target in an Ar–N_2_ atmosphere at room temperature. From atomic force microscopy, transmission electron microscopy and square resistance measurements we infer a film thickness of 8.2 nm and a deposition rate of 1.33 nm s^−1^. Transmission electron micrographs (Fig. 3c) on a reference device show that our NbTiN films are slightly thicker than those used in previous device generations^25^,^26^,^28^. This could explain the somewhat lower detection efficiency and dark count rate as compared with those reported in ref. 28, which relied on NbTiN films sputtered elsewhere. We anticipate that fine tuning of our NbTiN sputter recipe and film thickness will yield SSPD performance on a par with previous demonstrations.

After NbTiN film deposition we define electrode pads and alignment marks (Fig. 2a) for subsequent layers in electron-beam lithography using double-layer polymethyl methacrylate positive-tone resist. After development in methyl isobutyl ketone and isopropyl alcohol we deposit an 8-nm Ti adhesion layer and 150 nm gold (Au) in electron-beam evaporation followed by lift off in acetone. In a second high-resolution (100 kV) electron-beam lithography step the detector nanowires are patterned in negative-tone hydrogen silsesquioxane resist. Each detector pair is aligned separately to the Au alignment marks in the write-field of the respective device. After development in tetramethylammonium hydroxide-based developer the pattern is transferred to the NbTiN layer in a timed reactive-ion etching step employing tetrafluoromethane (CF4) chemistry. In a third and final electron-beam lithography step we expose the waveguide layer in positive-tone ZEP520A polymer resist. The patterns for each photonic circuit device are aligned to the same alignment marks used in the previous step for defining the respective nanowire detector pair. Following development in xylenes the waveguide patterns are transferred to the SiN film via carefully timed reactive-ion etching in fluoroform (CHF3). The resulting devices are shown in Figs 2 and 3.

## Additional information

**How to cite this article:** Schuck, C. *et al.* Quantum interference in heterogeneous superconducting-photonic circuits on a silicon chip. *Nat. Commun.* 7:10352 doi: 10.1038/ncomms10352 (2016).

## Supplementary Material

Supplementary Information Supplementary Figures 1-4, Supplementary Notes 1-3 and Supplementary References.
