# Supplementary material for: Quantum interference in heterogeneous superconducting-photonic circuits on a silicon chip
Source: Nat Commun. 2016 Jan 21;7:10352. doi: 10.1038/ncomms10352 (PMC4735806; doi:10.1038/ncomms10352)
Supplement: Supplementary Information — Supplementary Figures 1-4, Supplementary Notes 1-3 and Supplementary References. [file ncomms10352-s1.pdf]

Supplementary Information

Supplementary Figures

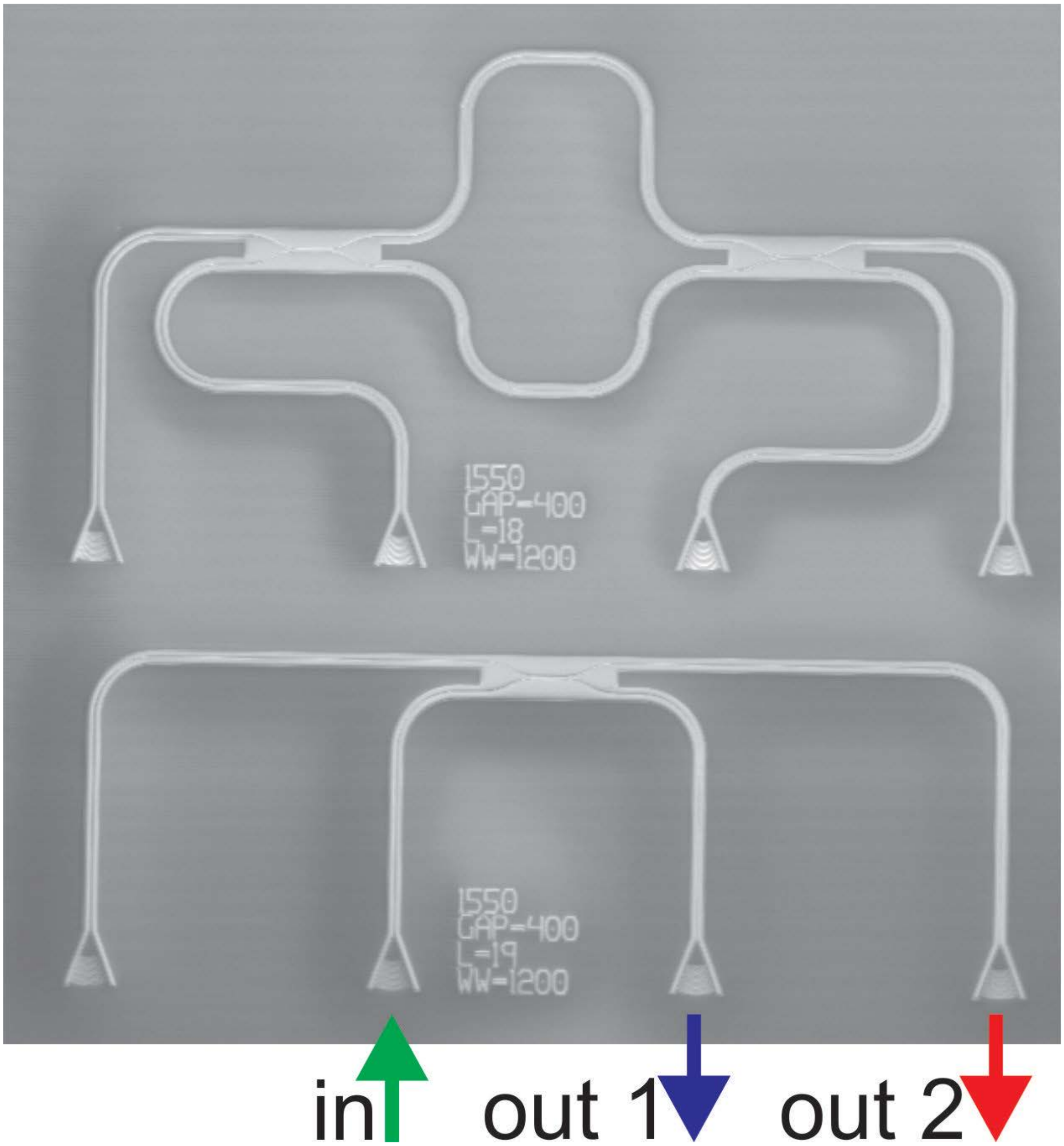

Supplementary Figure 1: Directional coupler calibration devices. Scanning electron micrograph of Mach-Zehnder interferometer (top) and beam splitter devices (bottom) to calibrate the directional coupler splitting ratio as a function of coupling length and gap between waveguides of given width and height. Laser light (λ =1550 nm) is injected at one input port and we detect the transmission at both output ports.

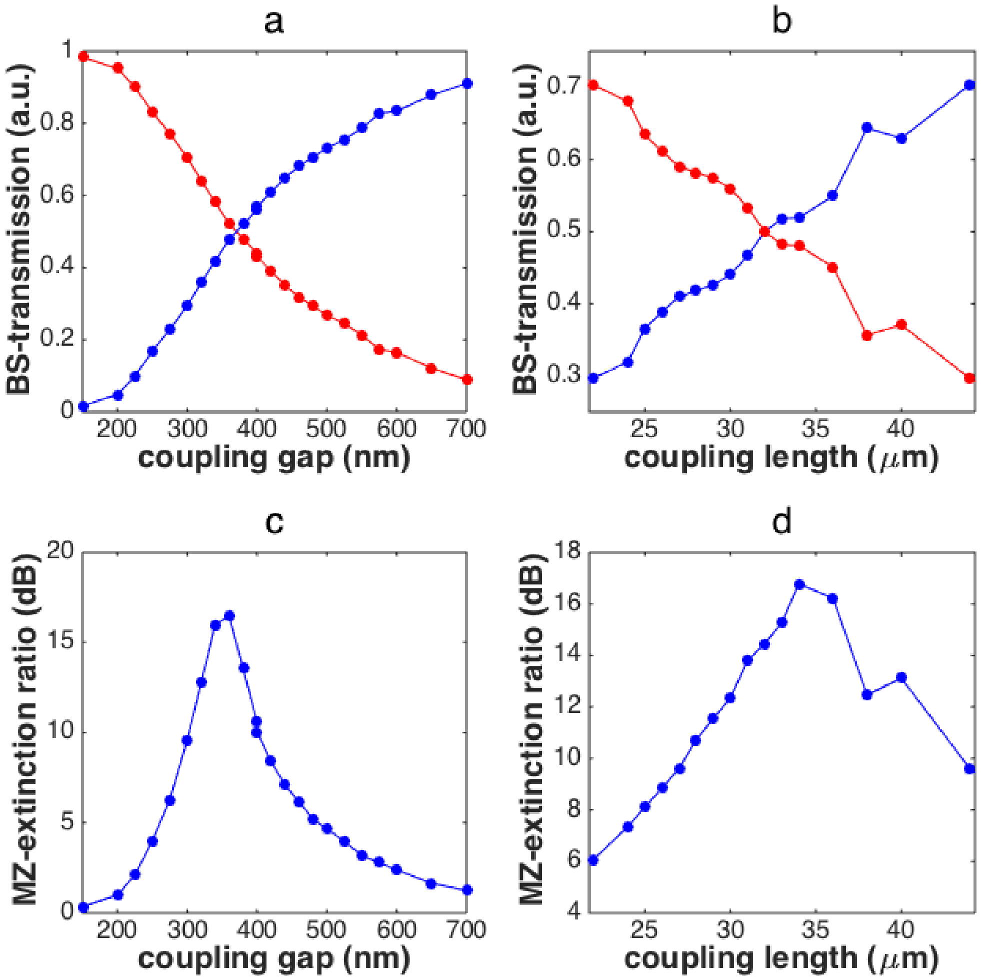

Supplementary Figure 2: Coupling gap and length calibration. For the beam splitter (BS) devices shown in supplementary figure 1 (bottom) we record the transmission at the output ports, a) for various coupling gaps 150-700 nm keeping the coupling length constant at 28 µm. 50:50 splitting is observed at 370 nm; b) for various coupling lengths 22-44 µm while keeping the coupling gap constant at 400 nm. 50:50 splitting is observed at 32 µm. For the Mach-Zehnder interferometer (MZ) devices shown in supplementary figure 1 (top) we record the extinction of interference fringes at the output ports, c) for various coupling gaps 150-700 nm keeping the coupling length constant at 28 µm. 50:50 splitting, corresponding to maximal extinction, is observed at 360 nm; d) for various coupling lengths 22-44 µm while keeping the coupling gap constant at 400 nm. 50:50 splitting, corresponding to maximal extinction, is observed at 34 µm.

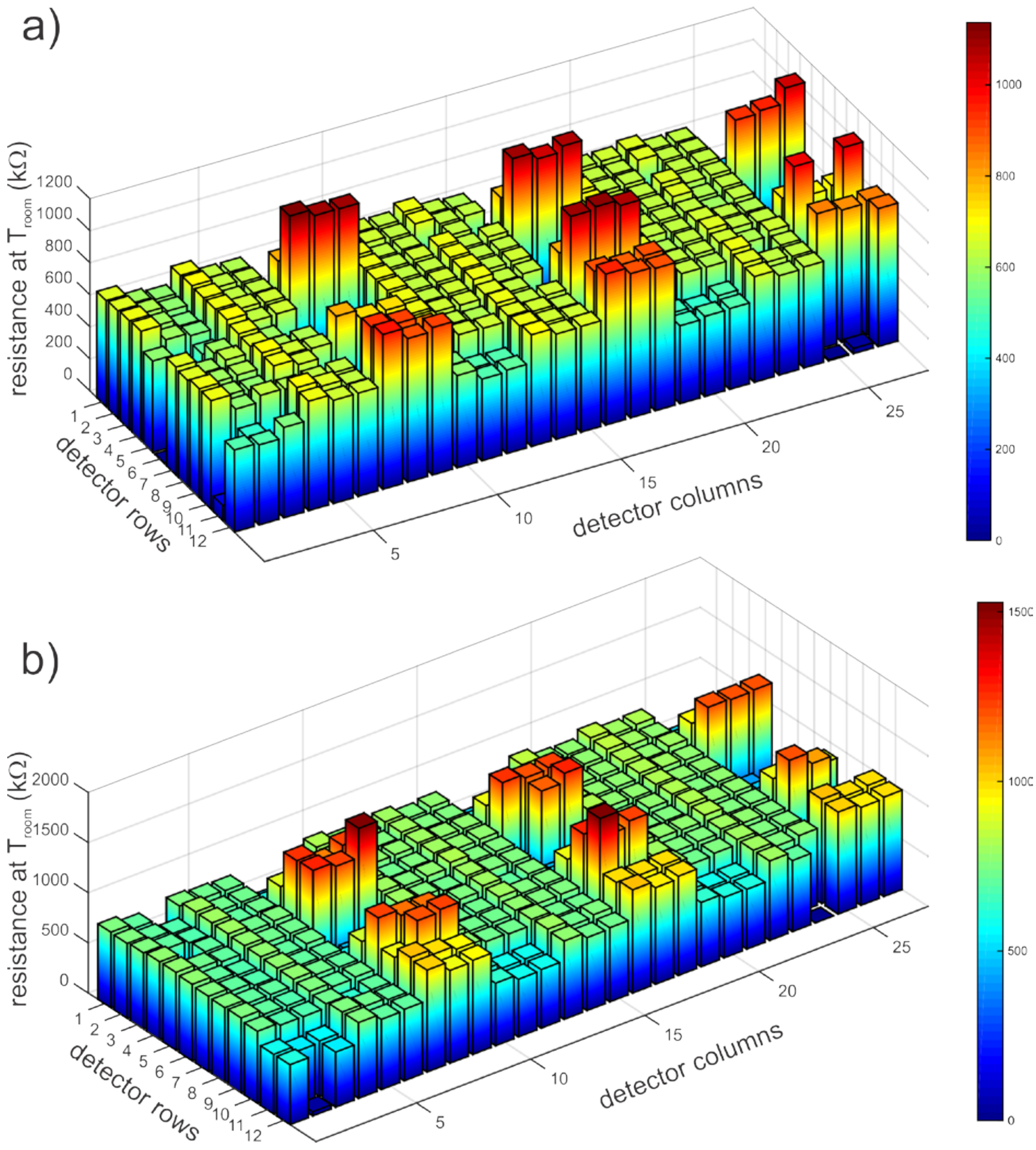

Supplementary Figure 3: Room temperature resistance of nanowire SSPDs. a) Room temperature resistance in kΩ (z-axis) for SSPDs on the chip used for the measurements presented in the main text (8.2 nm NbTiN film thickness), ordered in rows & columns on x-and y-axis. Nanowire width varies from 90 nm (e.g. row 1, column 7) to 25 nm (e.g. row 4, column 7); b) room temperature resistance measurement for SSPDs on a chip of similar device layouts but fabricated from 6.7 nm thin NbTiN film.

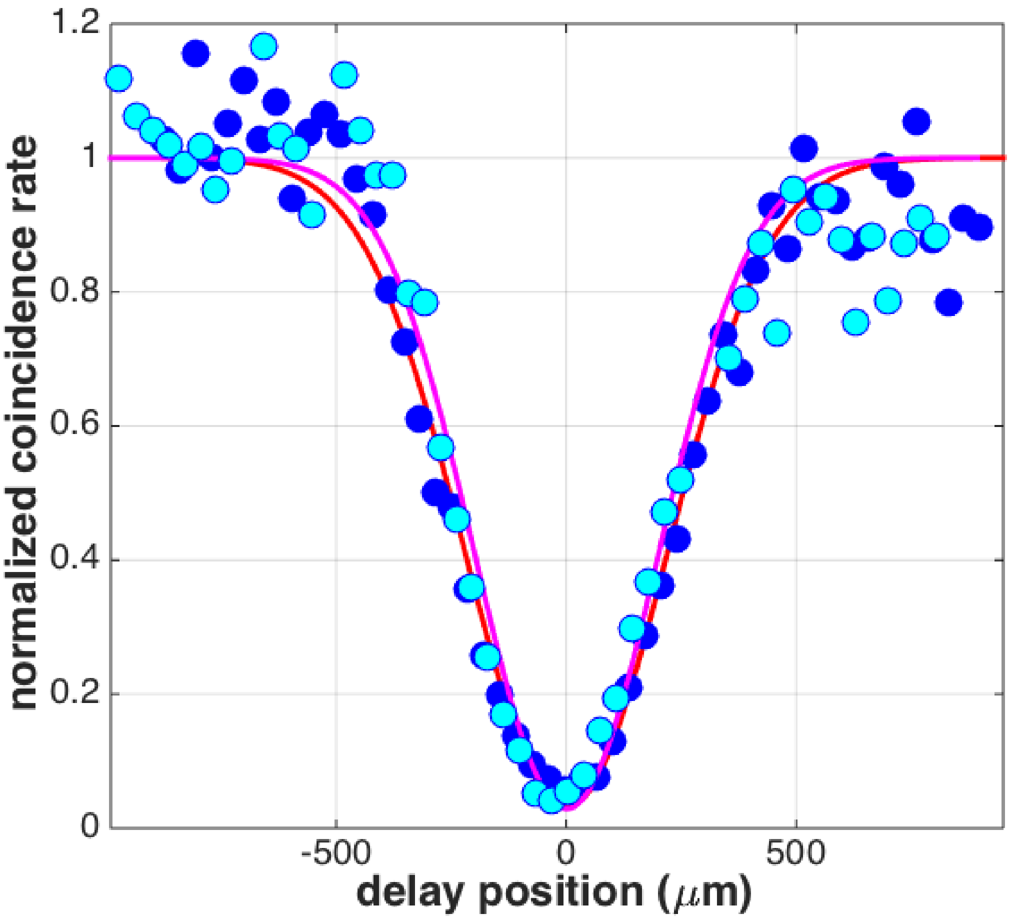

Supplementary Figure 4: HOM-interference measurement at different SPDC pump power. Cyan dots: coincidences measured at 3.5 mW power of the 775 nm SPDC-pump laser; magenta curve: fit to 3.5 mW measurement data. Visibility and width of the HOM-dip are found at 97.1% and $\mathbf{472 \pm 63\ \mu m}$, respectively; blue dots: coincidences measured at 10.5 mW power of the 775 nm SPDC-pump laser, for error bars see Fig. 4 in main text; red curve: fit to 10.5 mW measurement. The visibility and width of the HOM-dip are found at 96.9% and $\mathbf{518 \pm 41\ \boldsymbol{\mu}m}$, respectively. Data have been normalized to the respective coincidence rates at $\Delta\boldsymbol{\tau} \to \infty$, as determined from the fit to the 10.5 mW pump and the 3.5 mW pump data.

Supplementary Note 1

Calibration devices. For determining the coupling gap and length, L_c, which corresponds to 50:50 coupling between the two waveguides of a beam splitter, we fabricate Mach-Zehnder and beam splitter devices as shown in supplementary figure 1. For the beam splitter devices we inject a 1550 nm wavelength laser at one of the inputs and measure transmission through the device at both outputs of the directional coupler. For the Mach-Zehnder interferometer devices we scan the wavelength of the input laser around 1550 nm wavelength and record the resulting interference fringes at the outputs. Maximum extinction is expected for 50:50 splitting ratio. We fabricate devices of different coupling gap and width on a bare SiN on SiO_2 on Si chip, i.e. the material which is used as a substrate for fabricating the PIC-SSPD devices described in the main text. We scan the coupling gap and width around the design values determined from FEM simulations, i.e. 28 µm length for 400 nm gap (see main text).

The results of the calibration measurements are shown in supplementary figure 2. From scanning the coupling gap at (fixed) 28 µm coupling length we find 50:50 splitting for a 370 nm gap between waveguides (supplementary figure 2a). Similarly we observe maximal extinction (limited by the amplified spontaneous emission of the laser used in these measurements), corresponding to 50:50 coupling, in Mach-Zehnder interferometers consisting of two directional couplers both with 360 nm gaps and 28 µm coupling length by design (supplementary figure 2c). When fixing the gap at 400 nm and scanning the coupling length we find 50:50 splitting at 34 µm and 32 µm from the beam splitter transmission (supplementary figure 2b) and Mach-Zehnder interferometer extinction data (supplementary figure 2d), respectively. The mismatch between the design values determined by FEM simulations (see main text) and the measured values is likely due to a different device geometry of the fabricated devices with respect to the simplified design considered in FEM simulations.
Notably, we find very high device yield and similar grating coupler transmission across all devices (up to 6% of the input power through the device), which demonstrate the robustness of our fabrication recipe. These measurements are done in a separate setup at room temperature.

Based on the good agreement of the calibration data from BS and MZ devices we establish a gap of 400 nm and a coupling length of 33±1 µm as design parameters for the directional couplers used in the devices considered in the main text. Slight deviations from 50:50 splitting in PIC-SSPD devices can occur due to the additional processing steps required for SSPD fabrication. E.g. the etching of the NbTiN-layer can slightly reduce the underlying SiN film thickness (see Methods in main text) and thus result in a slight change of waveguide geometry in the coupling region, which in turn causes a slight splitting ratio offset from 50:50. We anticipate that this effect can be accounted for in future device generations.

Supplementary Note 2

Device yield. A typical chip design contains a large number of similar circuits (108 photonic circuits and 324 detectors in our case). For the chips used here we vary a large number of parameters across the devices on a chip, e.g. nanowire width of the SSPDs, grating coupler period, directional coupler gap and length, and other device geometry. To estimate fabrication yield of our circuits and detectors on a chip we perform device characterization at room temperature prior to cryogenic cooling because not all devices could be characterized at low temperature. The photonic waveguide devices are less critical than the nanowire detectors in terms of feature size but occupy a larger area. Optical transmission measurements yield similar results as compared to those presented in supplementary note 1 and indicate high yield (typically above 95%) of the nanophotonic waveguide circuits, as is routinely achieved for PICs (see also ref. [1]). On the other hand, the performance of the SSPDs is of particular interest for evaluating how device yield affects scalability of integrated quantum photonic circuits. We characterized twenty SSPDs at 1.7K and found for all of them similar efficiency compared to that reported in the main text. However, due to the relatively small number of devices studied at low temperature it is difficult to extract meaningful statistics for making statements about how device yield may affect scalability. Instead, we measure the room temperature resistance of all nanowire detectors on a chip and observed the values in supplementary figure 3 a. All detector-nanowires have the same length (40um) but the width is varied from 25 to 90nm. As expected from the design values we find the narrower nanowires to have higher room temperature resistance (red bars) and the wider nanowires with lower room temperature resistance (blue bars). For nanowires with width larger than 30 nm high yield is apparent from supplementary figure 3. For the example of the 198 nanowires with 50nm width (yellow-green bars) we identified 9 devices in supplementary figure 3 a, which clearly deviate from the median room temperature resistance at this width. At least three of these devices were scratched during chip handling in between fabrication steps, which suggest a yield of approximately 97%. To investigate how yield varies between fabrication runs we fabricated a second chip (with similar design but slightly different NbTiN-film thickness) and repeat the room temperature resistance screening. We find the data shown in supplementary figure 3 b, which even shows a slightly higher yield of 98.5% for 50 nm nanowire SSPDs.
However, we want to emphasize that, in general, similar room temperature resistance values are no guarantee for similar detector performance at low temperature but merely an indication. Future studies of fabrication yield of (large numbers of) superconducting nanowires, including careful characterization at cryogenic temperatures, are necessary to establish its influence on scaling integrated quantum photonic. However, our findings show no indications that the scalability of superconducting-photonic circuits would be limited by fabrication yield. In view of the possibility to take advantage of well controlled, automated processes commonly found in the semiconductor industry for future device generations we thus expect that functional large-scale integrated quantum photonic circuits are feasible.

Supplementary Note 3

Power dependence of HOM-interference. We measure HOM interference for different powers of the 775 nm pump laser to investigate the influence of higher order SPDC-processes on interference visibility. At 10.5 mW pump power we measure a coincidence rate of 4.2 Hz for photons arriving at the on-chip directional coupler with delay $\Delta\tau \rightarrow \infty$, as shown in Fig. 4a of the main text. The HOM-interference visibility was determined as 96.9% from a Gaussian fit to the data. We then reduce the 775 nm pump power for SPDC photon pair generation to 3.5 mW and repeat the two-photon interference measurement. In this case we determine a visibility of 97.1% from a Gaussian fit to the data. For direct comparison we show the data of each measurement in supplementary figure 4 normalized to the rate of uncorrelated coincidences (i.e. at $\Delta\tau \rightarrow \infty$) determined from the respective fit to the data.

The probability for generating *n* indistinguishable photon pairs in SPDC is given by [2]:

$$P_n = (1+n)\frac{\left(\frac{\mu}{2}\right)^n}{\left(1+\frac{\mu}{2}\right)^{n+2}} \tag{1}$$

where $\mu = 2\sinh^2(\chi t)$ is the average number of photon pairs produced during a time interval *t* for a given χ, which is proportional to the second order nonlinear susceptibility and the amplitude of the pump field [3]. The increase in the number of multi-photon-pair processes with pump power is thus almost linear for low μ and monotonically increases with pump power. In turn the visibility of two-photon interference is directly determined by the average number of generated photon pairs and reduces correspondingly with pump power.

The marginal difference of 0.2% in interference visibility observed here for a three-fold increase in pump power shows that the average number of generated photon pairs per *t*=256 ps is low enough such that higher order SPDC-processes do not contribute significantly to the measured coincidence rate (assuming bucket detectors, which do not resolve photon number) in this pump power regime.

Supplementary References

1. W. H. P. Pernice, C. Schuck, O. Minaeva, M. Li, G. Gol'tsman, A. V. Sergienko, H. X. Tang, "High-efficiency, ultrafast single-photon detectors integrated with nanophotonic circuits", Nat. Comm. **3**, 1325 (2012).
2. H. Takesue, K. Shimizu, “Effects of multiple pairs on visibility measurements of entangled photons generated by spontaneous parametric processes,” Opt. Commun. 283, 276–287 (2010).
3. D.F. Walls, G. J. Milburn, “Quantum Optics”, Springer, Berlin (2008).
